# Supplementary material for: Mechanism of Tumor Budding in Patient-Derived Metachronous Oral Primary Squamous Cell Carcinoma Cell Lines
Source: Int J Mol Sci. 2025 Apr 3;26(7):3347. doi: 10.3390/ijms26073347 (PMC11989605; doi:10.3390/ijms26073347)
Supplement: Supplementary file 1 [file ijms-26-03347-s001.zip › ijms-3523929-supplementary.pdf]

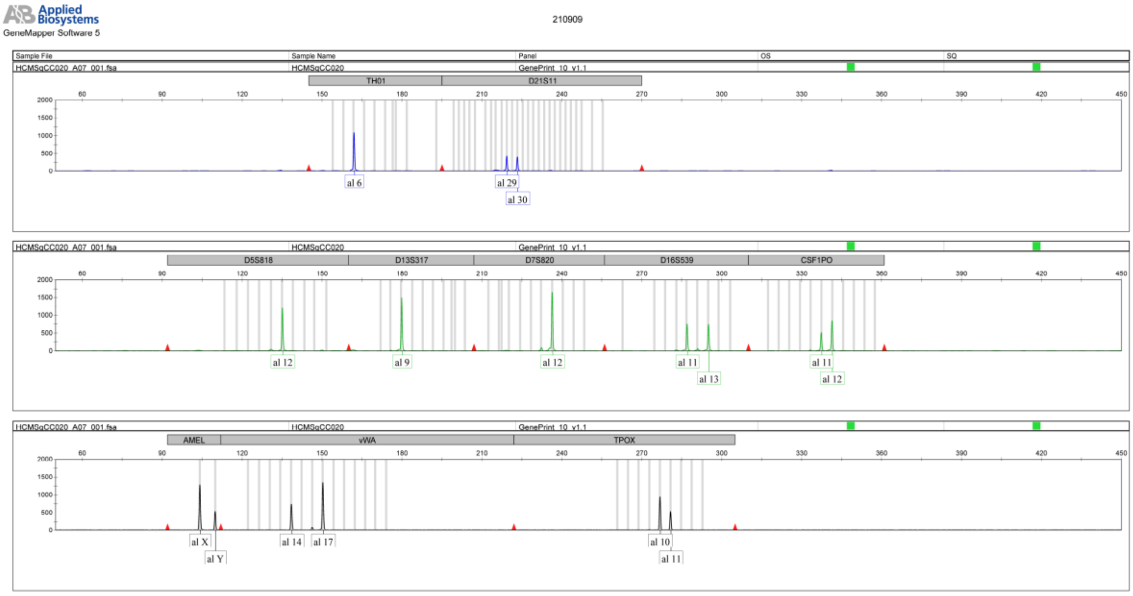


**Figure S1: STR analysis of 020 cells**

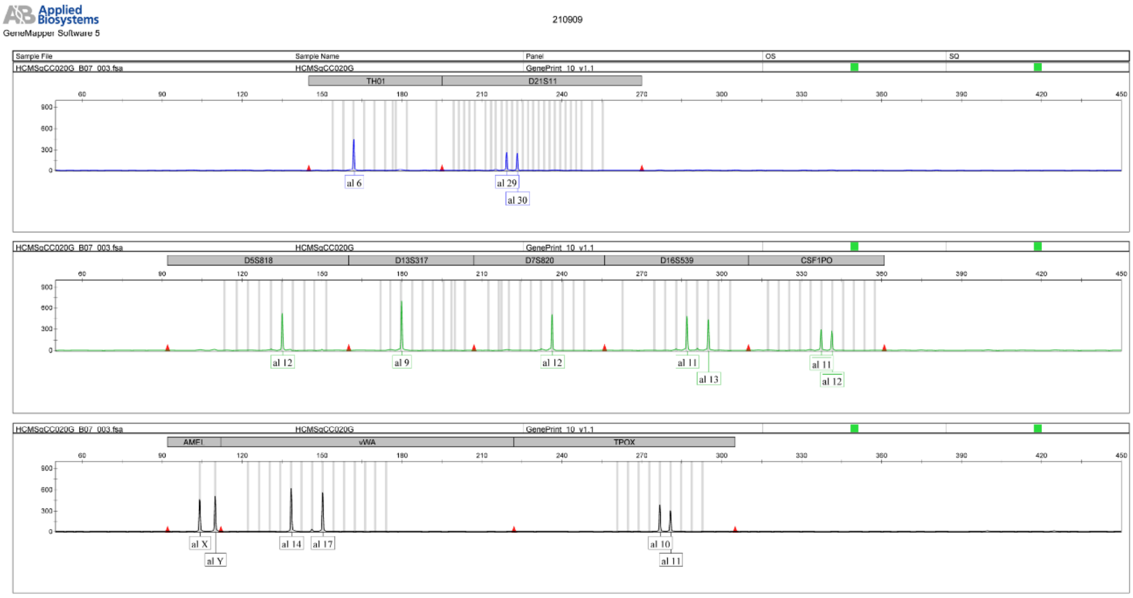


**Figure S2: STR analysis of 020G cells**

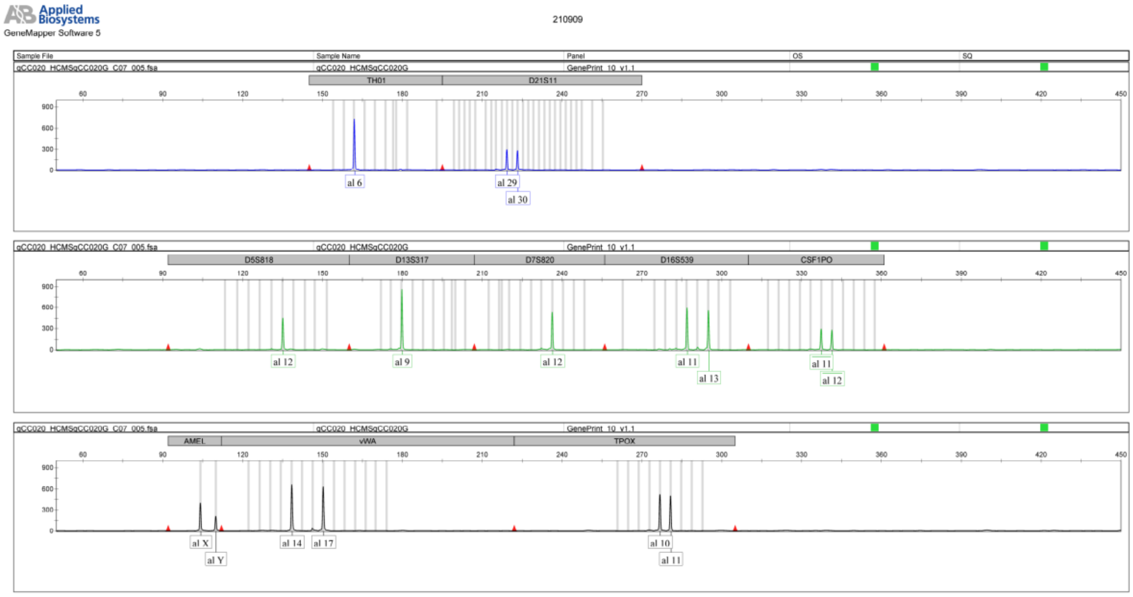


**Figure S3: STR analysis of patient blood-derived DNA**

**Table S1: Summary of STR Analysis**

| allele data | | | | | | |
|---|---|---|---|---|---|---|
| Locus | 020 cell DNA | | 020G cell DNA | | Blood DNA | |
| TH01 | 6 | | 6 | | 6 | |
| D21S11 | 29 | 30 | 29 | 30 | 29 | 30 |
| D5S818 | 12 | | 12 | | 12 | |
| D13S317 | 9 | | 9 | | 9 | |
| D7S820 | 12 | | 12 | | 12 | |
| D16S539 | 11 | 13 | 11 | 13 | 11 | 13 |
| CSF1PO | 11 | 12 | 11 | 12 | 11 | 12 |
| AMEL | X | Y | X | Y | X | Y |
| vWA | 14 | 17 | 14 | 17 | 14 | 17 |
| TPOX | 10 | 11 | 10 | 11 | 10 | 11 |

**Table S2: Antibodies for WB**

| Name | Company | Cat no. |
|---|---|---|
| Actin(C4) mouse mAb | SANTACRUZ | sc-47778 |
| E-cadherin(24E10) rabbit mAb | Cell Signaling Technology | 3195 |
| N-cadherin(13A9) rabbit mAb | SANTACRUZ | sc-7939 |
| β-catenin(H-102) rabbit mAb | SANTACRUZ | sc-7199 |
| Fibronectin rabbit mAb | Abcam | ab2413 |
| LAMC2 rabbit mAb | Abcam | ab96327 |
| SMAD2/3(D7G7) rabbit mAb | Cell Signaling Technology | #8685 |
| p-SMAD2/3(D27F4) rabbit mAb | Cell Signaling Technology | #8828 |
| YAP rabbit mAb | Cell Signaling Technology | 4912S |

Table S3: Primers for PCR

| Gene Name | Forward Primer (5'-3') | Reverse Primer (5'-3') | bp |
|---|---|---|---|
| *Zeb1* | TCCATGCTTAAGAGCGCTAGCT | ACCGTAGTTGAGTAGGTGTATGCCA | 78 |
| *Zeb2* | CAAGAGGCGCAAACAAGCC | GGTTGGCAATACCGTCATCC | 128 |
| *Snail1* | GGCGCACCTGCTCGGGGAGTG | GCCGATTCGCGCAGCA | 199 |
| *Snail2* | GGGGAGAAGCCTTTTTCTTG | TCCTCATGTTTGTGCAGGAG | 158 |
| *Twist1* | CGGACAAGCTGAGCAAGAT | CTGGAGGACCTGGTAGAGGA | 74 |
| *Twist2* | CGCAAGTGGAATTGGGATGC | CGATGTCACTGCTGTCCCTT | 114 |
| *MMP-2* | GGCTCATGCCTTCGCCCCAG | ACTCCCCATCGGCGTTCCCA | 122 |
| *MMP-9* | TGACAGCGACAAGAAGTG | CAGTGAAGCGGTACATAGG | 143 |
| *MT1-MMP* | CCCTATGCCTACATCCGTGA | TCCATCCATCACTTGGTTAAT | 532 |
| *LAMC2* | CTGAGTATGGGCAATGCCAC | GCTCTGGTATCAACCTTCTG | 452 |
| *GAPDH* | CAATGACCCCTTCATTGACC | GACAAGCTTCCCGTTCTCAG | 107 |
